# Integrative Deep Learning for Identifying Differentially Expressed (DE) Biomarkers

**DOI:** 10.1155/2019/8418760

**Published:** 2019-11-02

**Authors:** Jayeon Lim, SoYoun Bang, Jiyeon Kim, Cheolyong Park, JunSang Cho, SungHwan Kim

**Affiliations:** ^1^Department of Applied Statistics, Konkuk University, Seoul, Republic of Korea; ^2^Department of Data Science, Konkuk University, Seoul, Republic of Korea; ^3^Department of Statistics, Keimyung University, Daegu, Republic of Korea; ^4^Industry-University Cooperation Foundation, Konkuk University, Seoul, Republic of Korea

## Abstract

As a large amount of genetic data are accumulated, an effective analytical method and a significant interpretation are required. Recently, various methods of machine learning have emerged to process genetic data. In addition, machine learning analysis tools using statistical models have been proposed. In this study, we propose adding an integrated layer to the deep learning structure, which would enable the effective analysis of genetic data and the discovery of significant biomarkers of diseases. We conducted a simulation study in order to compare the proposed method with metalogistic regression and meta-SVM methods. The objective function with lasso penalty is used for parameter estimation, and the Youden J index is used for model comparison. The simulation results indicate that the proposed method is more robust for the variance of the data than metalogistic regression and meta-SVM methods. We also conducted real data (breast cancer data (TCGA)) analysis. Based on the results of gene set enrichment analysis, we obtained that TCGA multiple omics data involve significantly enriched pathways which contain information related to breast cancer. Therefore, it is expected that the proposed method will be helpful to discover biomarkers.

## 1. Introduction

With the development of base sequence measurement tools, it has become possible to process a large amount of gene data at high speed. This has enabled the accumulation of large amounts of genetic data and facilitated the development of various analytical techniques and tools for analyzing such accumulated data. The use of high-level analysis techniques and tools is required to interpret large quantities of genetic data. For this reason, it is very important to analyze such genetic data using the most advanced computing methods and mathematical and statistical techniques available for quickly processing genetic big data.

Furthermore, it is important to discover the significant genes associated with diseases in various genetic data. Genetic big data contain sparse genes or proteins relating to the etiology of diseases, which sometimes could be difficult to identify. These significant genes are called biomarkers. Biomarkers are indicators that could distinguish between normal and morbid conditions, predict and evaluate treatment responses, and objectively measure certain cancers or other diseases. Moreover, biomarkers could objectively assess the responses of drugs to normal biological processes, disease progress, and treatment methods. Some biomarkers also serve as disease identification markers that could detect early changes of health conditions.

In this paper, we propose the integrative deep learning for identifying biomarkers, a deep learning algorithm with a consolidation layer, and compare it with other machine learning methods based on a simulation along with real data (TCGA) analysis. Artificial neural networks (ANNs) are one of the main tools used in machine learning. Artificial neural networks (ANNs) are computing systems which are inspired by the biological neural networks of animal brains. An ANN consists of a set of processing elements, also known as neurons or nodes, which are interconnected [1]. Artificial neural networks (ANNs) which consist of an input layer, more than one hidden layers, and an output layer are called as deep neural networks. Training them is called as deep learning. In this study, we use a single hidden layer. Deep learning is widely applied in bioinformatics area. For example, Lee et al. [2] employed deep learning neural networks with features associated with binding sites to construct a DNA motif model. In addition, Khan et al. [3] developed a method of classifying cancers to specific diagnostic categories based on their gene expression signatures using artificial neural networks (ANNs).

In our method, the learning process proceeds in the following order: first, feedforward calculation is performed from the input layer to the output layer by using the weights in each layer. At this time, when the signal is passed from the input layer to the hidden layer and from the hidden layer to the output layer, the activation function is used to determine the intensity of the signal. The backpropagation algorithm is then used to reduce the difference between the output and actual values, starting from the output layer. The gradient descent optimization algorithm is used to modify the weights and minimize the errors. The feedforward and backpropagation algorithms are repeatedly carried out as many times as necessary for learning, and the learning is performed by updating the weights, which are the parameters used in each step. The algorithms are explained further in detail in Section 2.2.

Data analysis for single omics data is limited to correlation analysis, and it mostly represents the result of the reaction process rather than the cause process [4]. For this reason, in this study, we used integrated multiple omics data that integrate single omics data. As a method for omics data integration, the network biology approach emphasizes the interactions of genomic data such as genes and proteins. This provides a framework for data integration to analyze disease, and it is an approach to modeling genomic data [5]. In contrast to the integrated omics data, the size of each sample is limited for single omics data, and thus common markers were seldom found in studies on the same cancer. By integrating the data and increasing the sample size, more reliable biomarkers can be found than those found through the use of single omics data alone [6]. Various statistical methodologies are applied to analyze an integrated omics dataset, including the application of a group lasso penalty [7] and the proposed method for the lasso model [7].

Meta-analysis is another method that is useful for analyzing omics data. Meta-analysis is a method that involves objectively and quantitatively aggregating the results of many studies involving the same or similar subjects. Since life phenomena usually occur through interactions with other organs, the use of meta-analysis for omics data is very effective. A meta-analysis combines not only individual hypotheses but also the associated assumptions for significant results [8]. Furthermore, the MetaKTSP predictive model with ranking-based algorithms shows excellent performance in detecting biomarkers [9]. In addition, Kim et al. [10] proposed MetaPCA based on a statistical method. Various methods of machine learning analyses have been proposed to analyze data accumulated in large quantities.

Machine learning analysis methods have widely been used to analyze data in a variety of fields of biology [11]. Moreover, the analytical methods of machine learning and related algorithms for processing such genetic big data have been developed. For example, see the support vector machine (SVM) [12], meta-SVM and metalogistic regression [7], and various machine learning models [13]. In addition, the MLSG (machine learning system genomics) approach, in which a machine learning method is combined with biological methods for analyzing the multiple integrated omics data, is more useful than an approach using single omics data alone [14]. The development of various machine learning methods has enabled the modeling of genes related to diseases, and they have obtained meaningful analysis results. Microarray and next generation sequencing (NGS) data are important for finding useful molecular patterns, and more studies on gene expression data are needed to identify the biomarkers associated with cancer [15]. Gene expression data could also be used to identify various diseases and potentially cancerous genes [16]. We could also see that gene expression data have a strong influence on identifying biomarkers [17].

## 2. Methods

### 2.1. Experimental Design

RStudio was used for analysis along with R packages such as coda, MASS, foreach, iterators, parallel, doMC, e1071, MCMCpack, penalized, and glmnet. Deep learning, a method of machine learning, was used to classify gene expression data and other data related to breast cancer. A simulation study was conducted before analyzing the actual breast cancer data. We randomly generated data of 80 integrated layers, consisting of 16 signal genes (three in each of the two clusters) and 64 nonsignal genes. For the random study, we generated data with no signal gene. The *β* values of the integrated layer for the nonsignal genes will be close to zero, whereas the *β* values of the signal genes will not be close to zero. In addition, several values of *σ* and *λ*, lasso penalty, were used during the data generation. As the *λ* value becomes very large, *β* values of all of the layers converge to zero. Finally, we obtained the value of the Youden J index using the actual values and the predicted values from the algorithm execution. The value of the Youden J index can be used to evaluate the performance of deep learning.

### 2.2. Structure

**Figure 1:**
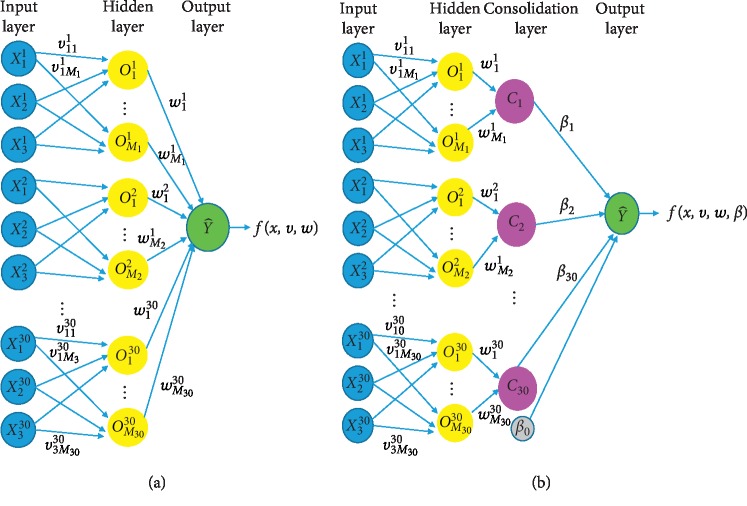
(a) Deep learning structure and (b) deep learning structure with consolidating layer.

It is common that the artificial neural networks (ANNs) are composed of fully connected layers through the architecture. Yet our proposed model, by extension, aims at not only prediction but also variable selection by means of penalization to particular gene modules. To this end, the model purposely accommodates the weights at the last layer, whose module counts are as the number of genes at the consolidating layers. The deep learning structure with a consolidation layer is constructed by adding a consolidation layer to the usual deep learning structure, as shown in Figure 1. Each consolidation layer combines the results from three genes of the datasets at the same position. The superscript is the gene number while the subscript is the dataset number. The initial value of each weight starts with 1.

#### 2.2.1. Issues regarding Initial Weights

In deep learning, it is important to set initial weight properly. There is no guarantee that objective function is convex due to the nature of deep learning algorithm. Moreover, local minima might exist at several points. If we start from the arbitrary point, there is no guarantee that it converges to global optimum, and it even cannot converge anywhere. Therefore, rather than arbitrary initial weight, we suggest more systemic method that sets initial weight:

1. Set initial weight *w*, *v* to 1.
2. Generate *C*^*k*^=∑_*m*=1_^*M*_*k*_^*w*_*m*_^*k*^*O*_*m*_^*k*^ for each integrated *X*, and set all *w*_*m*_^*k*^ to 1 where *m*=1,…, *M*_*k*_, *v*_*m*_ to 1 in *O*_*m*_^*k*^=*a*_*m*_(*x* · *v*_*m*_).
3. Make design matrix *C*_(*N* × (*K*+1))_ from generated *K* vectors and 1 vector for bias term.
4. Due to the nature of omics data, we often cannot fit the linear regression as the number of variables (*p*) might be greater than the number of samples (*n*). Therefore, we fit the Ridge regression by generated design matrix *C*:

(1) $$\begin{array}{c} {\hat{\beta }}^{\text{Ridge}}={\left(C^{T}C+\lambda I\right)}^{-1}C^{T}Y. \end{array}$$

(5) Use ${\hat{\beta }}_{0}$ for initial *β*_0_, $\left({\hat{\beta }}_{1},\ldots ,{\hat{\beta }}_{k}\right)$ for initial weights.

#### 2.2.2. Feedforward

(1) *Input to Hidden Layer*. The values of the hidden layers are calculated from the input values as follows: first, let *K* be the number of genes in each dataset and let *M*_*k*_ be the number of hidden nodes in the *k*-th gene. Then, the values of the hidden layers can be calculated by
  (2) $$\begin{array}{c} O_{m}^{k}=\sigma_{*}\left(a_{m}^{k}\right), \end{array}$$
  where
  (3) $$\begin{array}{c} a_{m}^{k}=\overset{P_{k}}{\underset{p=1}{\sum }}v_{pm}^{k}x_{p}^{k},\;k=1,\ldots ,K,\;m=1,\ldots ,M_{k},\;p=1,\ldots ,P_{k}, \end{array}$$
  and *σ*_*∗*_ is ReLU (rectified linear unit) activation function.
(2) *Hidden to Consolidation*. Using the values in (1), the values of the integrated layer are calculated by
  (4) $$\begin{array}{c} C_{k}=\overset{M_{k}}{\underset{m=1}{\sum }}O_{m}^{k}w_{m}^{*k}=O_{m}^{k}\frac{w^{k}}{\left\|w^{k}\right\|}. \end{array}$$
(3) *Consolidation to Output*. Using the values in (2), the output value is calculated by
  (5) $$\begin{array}{c} \hat{y}=\beta_{0}+\overset{K}{\underset{k=1}{\sum }}\beta_{k}C_{k}, \end{array}$$
  and the predicted value is calculated by
  (6) $$\begin{array}{c} \hat{f}=\frac{1}{1+\exp\left(-\hat{y}\right)}. \end{array}$$
(4) *Objective Function*. The objective function for parameter estimation is given by
  (7) $$\begin{array}{c} R\left(\theta \right)=-\frac{1}{N}\overset{N}{\underset{i=1}{\sum }}\left\{y_{i}\log\;f_{i}+\left(1-y_{i}\right)\log\left(1-f_{i}\right)\right\}, \end{array}$$
  where
  (8) $$\begin{array}{c} f_{i}=\frac{1}{1+\exp\left(-\beta_{0}-\sum_{k=1}^{K}\beta_{k}C_{k}^{i}\right)}. \end{array}$$
  We used the objective function with lasso penalty given by
  (9) $$\begin{array}{c} R^{\lambda }\left(\theta \right)=-\frac{1}{N}\overset{N}{\underset{i=1}{\sum }}\left\{y_{i}\log\;f_{i}+\left(1-y_{i}\right)\log\left(1-f_{i}\right)\right\}+\lambda \overset{K}{\underset{k=1}{\sum }}\left|\beta_{k}\right|. \end{array}$$

#### 2.2.3. Backpropagation

1. *β*_*k*_. We calculate the first and second partial derivatives of *R*^*λ*^ with respect to *β*_*k*_. The first partial derivative of *R*^*λ*^ with respect to *β*_*k*_ is given by

(10) $$\begin{array}{c} \frac{\partial R^{\lambda }}{\partial \beta_{k}}=\frac{1}{N}\overset{N}{\underset{i=1}{\sum }}\left(f_{i}-y_{i}\right)C_{k}^{i}+\text{sign}\left(\beta_{k}\right)\lambda . \end{array}$$

  The second partial derivative of *R*^*λ*^ with respect to *β*_*k*_ is calculated as follows. The second partial derivative of *R*^*λ*^ with respect to *β*_*k*_ is given by

(11) $$\begin{array}{c} \frac{\partial^{2}R^{\lambda }}{\partial^{2}\beta_{k}^{2}}=\frac{1}{N}\overset{N}{\underset{i=1}{\sum }}f_{i}\left(1-f_{i}\right){\left[C_{l}^{i}\right]}^{2}. \end{array}$$

  Since sign(*β*_*k*_) is not differentiable at 0, we use a differential sigmoid function to approximate it. Let *z*(*β*) be a sigmoid function with scale parameter *s* given by

(12) $$\begin{array}{c} z\left(\beta \right)=2\frac{\exp\left(\beta /s\right)}{\left[1+\exp\left(\beta /s\right)\right]}-1, \end{array}$$

  where *s* is a small number, say 10^−3^. Since as *s*⟶0, *z*(*β*)⟶1 for *β* > 0, and *z*(*β*)⟶−1 for *β* > 0, we note that *z*(*β*) is approximately equal to sign(*β*) for small *s*. Since the first partial derivative of *z*(*β*) with respect to *β* is given by

(13) $$\begin{array}{c} z^{\prime }\left(\beta \right)=2s\frac{\exp\left(\beta /s\right)}{\left[1+\exp\left(\beta /s\right)\right]}\cdot \frac{1}{\left[1+\exp\left(\beta /s\right)\right]}, \end{array}$$

  and the second partial derivative of *R*^*λ*^ with respect to *β*_*k*_ is approximated by

(14) $$\begin{array}{c} \frac{\partial^{2}R^{\lambda }}{\partial^{2}\beta_{k}^{2}}\approx \frac{1}{N}\overset{N}{\underset{i=1}{\sum }}f_{i}\left(1-f_{i}\right){\left[C_{l}^{i}\right]}^{2}+\lambda z^{\prime }\left(\beta_{k}\right). \end{array}$$

  From the second partial derivative, the updated value of *β*_*k*_ is given by

(15) $$\begin{array}{c} {\tilde{\beta }}_{k}⟵\beta_{k}-\frac{\sum_{i}^{N}\left(f_{i}-y_{i}\right)C_{k}^{i}+N\lambda \text{sign}\left(\beta_{k}\right)}{\sum_{i}^{N}f_{i}\left(1-f_{i}\right)C_{k}^{i}+N\lambda z^{\prime }\left(\beta_{k}\right)}. \end{array}$$

  In updating *β*_*k*_, we use the idea from the Newton–Raphson method.
(2) *β*_0_. The first partial derivative of *R*^*λ*^ with respect to *β*_0_ is given by

(16) $$\begin{array}{c} \frac{\partial R^{\lambda }}{\partial \beta_{0}}=\frac{1}{N}\overset{N}{\underset{i}{\sum }}\left(f_{i}-y_{i}\right), \end{array}$$

  and the updated value of *β*_0_ is given by

(17) $$\begin{array}{c} {\tilde{\beta }}_{0}⟵\beta_{0}-\eta \frac{\partial R^{\lambda }}{\partial \beta_{0}}. \end{array}$$

(3) *w*_*m*_^*k*^. The first partial derivative of *R*^*λ*^ with respect to *w*_*m*_^*k*^ is given by

(18) $$\begin{array}{c} \frac{\partial R^{\lambda }}{\partial w_{m}^{k}}=\frac{1}{N}\overset{N}{\underset{i=1}{\sum }}\left(f_{i}-y_{i}\right)\beta_{k}\left\{\frac{1}{\left\|w^{k}\right\|}\left[O_{mi}^{k}-\frac{w_{m}^{k}}{{\left\|w^{k}\right\|}^{2}}\overset{M_{k}}{\underset{s=1}{\sum }}w_{s}^{k}O_{s}i^{k}\right]\right\}, \end{array}$$

  and the updated value of *w*_*m*_^*k*^ is given by

(19) $$\begin{array}{c} w_{m}^{\tilde{k}}⟵w_{m}^{k}-\eta \frac{\partial R_{\lambda }}{\partial w_{m}^{k}}. \end{array}$$

(4) *v*_*pm*_^*k*^. The first derivative of *R*^*λ*^ with respect to *v*_*pm*_^*k*^ is given by

(20) $$\begin{array}{c} \frac{\partial R^{\lambda }}{\partial v_{pm}^{k}}=\frac{1}{N}\overset{N}{\underset{i=1}{\sum }}\left(f_{i}-y_{i}\right)\beta_{k}\frac{w_{m}^{k}}{\left\|w_{m}^{k}\right\|}\left[\sigma_{*}^{\prime }\left(a_{mi}^{k}\right)x_{\pi }^{k}\right], \end{array}$$

  and the updated value of *v*_*pm*_^*k*^ is given by

(21) $$\begin{array}{c} v_{pm}^{\tilde{k}}⟵v_{pm}^{k}-\eta \frac{\partial R^{\lambda }}{\partial v_{pm}^{k}}. \end{array}$$

## 3. Simulation Study

### 3.1. Datasets

**Figure 2:**
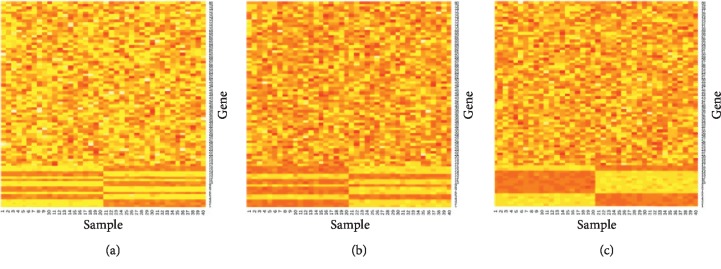
Heatmap of three datasets for *σ*=0.1.

**Figure 3:**
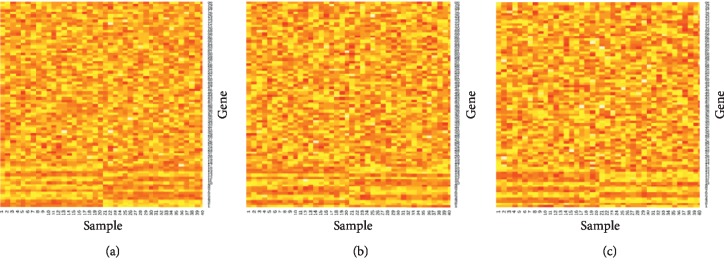
Heatmap of three datasets for *σ*=0.3.

The data generation was conducted as follows: MVN(0, *σ*^2^*I*), multivariate normal distribution with mean 0 and covariance matrix *σ*^2^*I*, was used to generate nonsignal genes, and MVN(*μ*, *σ*^2^Σ) was used to generate signal genes. 16 genes have signals, and the remaining 64 genes do not have signals. We generated three datasets and then put them in the input layer. When random study data were included, we generate datasets which have no signal genes. The three datasets for *σ*=0.1 are shown in Figure 2. In addition, Figure 3 shows the heatmap for *σ*=0.3 so that we can note that the heatmap for a larger *σ* has more noise. In light of simulated data, we randomly sampled training (70%) and testing (30%) data of three methods (i.e., meta-SVM, metalogistic regression, and integrative deep learning) in accordance with predetermined experiment designs and applied to the identical data to guarantee fair comparison.

### 3.2. Simulation Results

Tables 1–3 show the simulation results of metalogistic regression, meta-SVM, and the integrative deep learning. Sensitivity is the correct classification rate for signal genes, specificity is the correct classification rate for nonsignal genes, and the Youden J index is sensitivity + specificity − 1.  Table 1 shows the simulation results when no random study was included, and Tables 2 and 3 show the results when 1 and 2 random studies were included, respectively. Based on the Youden J index, integrative deep learning performs better than meta-SVM when the variance of the data is higher and performs better than metalogistic for all variances (*σ*) considered. Furthermore, the proposed method has more balanced values of sensitivity and specificity than metalogistic and meta-SVM.  Table 1 shows the simulation results of the case with no random studies included. We noted that integrative deep learning is better than meta-SVM, except for when data are sampled with low variance (for *σ* = 0.1, 0.9859 for meta-SVM, and 0.5973 for integrative deep learning). Meta-SVM performs quite well on that condition, but the Youden J index decreases radically as the variance of the data (*σ*) increases (0.1597, 0.1491, 0.5100, and 0.0120 for meta-SVM; 0.4427, 0.4119, 0.3666, and 0.3427 for integrative deep learning). Metalogistic results in a low Youden J index due to the low sensitivity. In comparison, integrative deep learning has a good balance between sensitivity and specificity, and the Youden J index decreases relatively slowly. Tables 2 and 3 show the simulation results of the inclusion of one and two random studies, respectively. When random studies were included, the results are similar to those with no inclusion of a random study. In Table 2 (the inclusion of one random study), meta-SVM performs better than deep learning when the variance of data is low (0.8045 and 0.6345 for meta-SVM; 0.6052 and 0.4114 for integrative deep learning). However, the Youden J index decreases radically as the variance increases (0.3435, 0.0138, and 0.3354 for meta-SVM; 0.3697, 0.3375, and 0.3411 for integrative deep learning). Metalogistic still has a low Youden J index. In Table 3 (the inclusion of two random studies), integrative deep learning performs better than meta-SVM, except for when data are sampled with low variance (*σ* = 0.1), and it also performs better than metalogistic for all experiment scenarios. Meta-SVM even results in zero for certain values of *σ*. All together, integrative deep learning always performs better than metalogistic, yet, meta-SVM performs better, particularly when data are sampled with low variance. However, the results of meta-SVM lack stability for the variance of the data, as they decrease radically. By contrast, integrative deep learning performs stably. Based on these results, we can identify that the integrative deep learning method is robust for the variance of the data. All simulations were repeated 30 times. This feature can be powerful for discovering significant biomarkers.

### 3.3. Tuning *λ* Values

**Figure 4:**
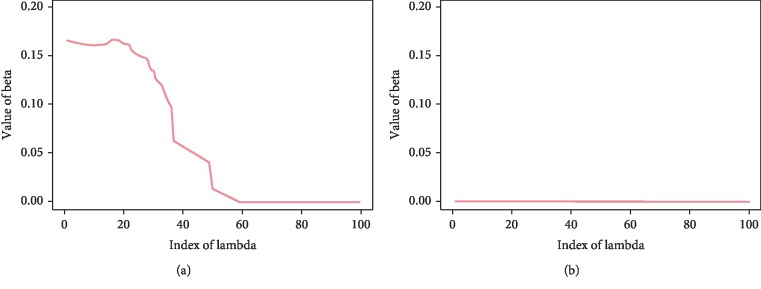
Estimated *β* values for (a) signal genes and (b) nonsignal genes.

The magnitudes of the estimated signal *β* changes nonzero to zero values after some *λ* values, while the magnitudes of the estimated nonsignal *β* are approximately zero for all of the *λ* values as shown in Figure 4. In all cases, the estimated *β* values converge to zero as *λ* becomes increasingly large. We can find the optimal *λ* value that distinguishes between signal and nonsignal through cross validation.

## 4. Applications to Real Genomic Data

In this section, we apply integrative deep learning methods to real examples of breast cancer expression profiles provided by The Cancer Genome Atlas (TCGA) including mRNA, copy number variation (CNV), and epigenetic DNA methylation (http://cancergenome.nih.gov/; 300 samples of estrogen receptor binary outcome (i.e., ER+ and ER−)). There are three types of data: mRNA, methylation, and CNV. We preprocessed the TCGA data according to Kim et al. [18]. The data obtained from TCGA data portal contain CNV for 23,235 genes, methylation levels of 22,529 probes, and mRNA expression levels for 17,814 genes. We filtered out genes with low-expressed (mean <0.9) or noninformative (standard deviation < 0.85) features in the mRNA expression data, and thereby, we obtained 1,345 methylation probes and 828 CNV genes by matching 828 mRNA gene symbols.  Table 4 presents the data descriptions. Each type of data consists of 234 controls and 66 cases for a total of 300 samples. We align three genomic data by the common cohort in the context of vertical integration. For methylation data, we selected only one gene data among the same gene data by leaving the one with the greatest IQR (interquartile range).

### 4.1. Results

We applied gene set enrichment analysis to TCGA breast cancer data in order to determine whether our identified gene sets are consistent with the underlying biological pathways from the KEGG (2016) database (https://www.genome.jp/kegg/pathway.html). The result of gene set enrichment analysis is described in Kim et al. [18] identified that TCGA multiple omics data are significantly enriched in the ABC transporter pathways, which is already well known to be correlated to breast cancer mechanisms and particularly related to estrogen receptors and drug resistance. Similarly, we found that our selected gene set enriched the ABC transporter pathways from the KEGG database. We also found that it enriched CAMs (cell adhesion molecules) pathways. According to Saadatmand et al. [19], CAM (cell adhesion molecule) pathways are known to play an important role in the process of metastasis. CAMs are a subset of proteins located on the cell surface. The major cause of breast cancer death is metastasis. The prognostic values of the tumor expression of N-cadherin, E-cadherin, carcinoembryonic antigen (CEA), and epithelial CAM (Ep-CAM) were evaluated in patients with breast cancer. There are four subfamilies of CAMs: cadherins, integrins, selectins, and immunoglobulins, such as carcinoembryonic antigen (CEA). N-cadherin and E-cadherin belong to cadherins, and CEA belongs to immunoglobulins. Ep-CAM is a type of CAM but does not belong to any of the four subfamilies mentioned above. Among these, combining E-cadherin and CEA tumor expression provides a prognostic parameter with high discriminative power that is a candidate tool for predicting prognosis in breast cancer. In addition, Li and Feng [20] identified CAMs in the paradigm of breast cancer. In breast cancer, the reduced expression of E-cadherin has been reported in approximately 50% of invasive ductal carcinomas, whereas invasive lobular carcinomas showed complete loss of E-cadherin expression in nearly 90% of cases and have also been shown to contribute to metastasis. Additionally, Li and Feng [20] stated that research on immunoglobulins in breast cancer has identified several members that are upregulated during cancer progression and that are potentially associated with an unfavorable prognosis and CEA comes under that. Overall, the results that we observe are consistent with existing biological truth. Thus, integrative deep learning is found to be an efficient method for discovering significant biomarkers of disease.

### 4.2. Gene Networks

**Figure 5:**
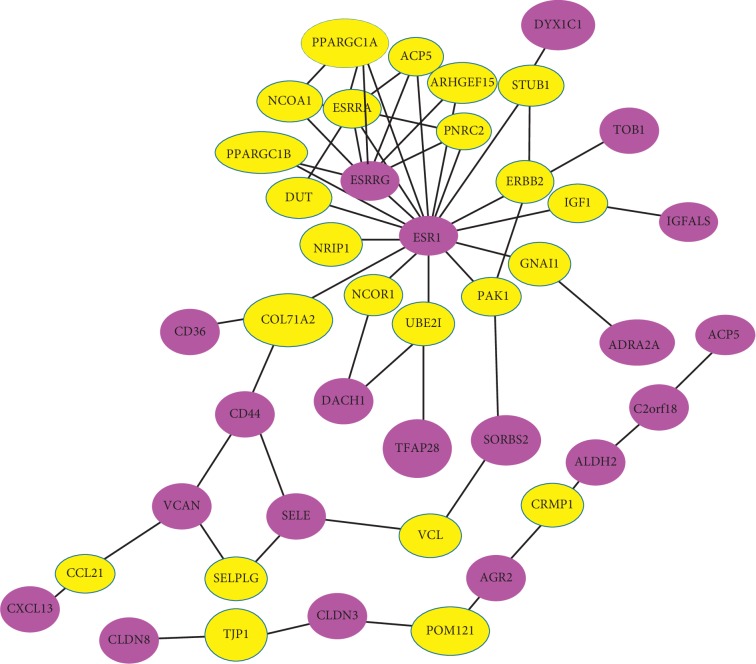
Gene networks that display the relationships among significant genes.

*NetBox* is an analytic software well suited to detect connecting genes to a network, identifying statistically significant linker genes on the basis of four public data sources: NCI-Nature Pathway Interaction Database, Human Protein Reference Database, MSKCC Cancer Cell Map (http://www.mskcc.org/), and Reactome Pathway Database.  Figure 5 shows the gene networks, which present the relationships among significant genes, via *NetBox*. The violet nodes are the selected linker genes out of the 59 genes listed in Table 5. The yellow nodes indicate linker genes that are not present in the original input list but are significantly connected to members of the input list. In gene networks, it is notable that the ESR1 gene is connected to many other genes. It appears that ESR1 plays an important role in these networks. According to Clatot et al. [21], ESR1 mutations are prominent in breast cancer. In particular, ESR1 mutations have recently emerged as a key mechanism of AIs (aromatase inhibitors) resistance in ER + metastatic breast cancer. Additionally, the ESRRG gene is also prominent in gene networks. Madhavan et al. [22] identified that ESRRG signaling is associated with poor distant metastasis-free survival in ER + as well as tamoxifen-treated breast cancer. Overall, our gene networks consist of genes related to breast cancer. We also construct gene networks by using software called *String* tool. (see Supplementary [Supplementary-material supplementary-material-1]).

## 5. Discussion

In order to predict the disease, it is crucial to identify genes related to the disease. In the analysis of such gene data, a machine learning method capable of processing genetic big data and statistical knowledge capable of interpreting these data are required. As technology advances, genomic data generation tools become more diverse and data generation speeds up faster. For this reason, a higher level of analysis is required. Moreover, it is generally known that combining multiple studies can improve statistical power and provide validated conclusions. In addition to metalogistic and meta-SVM, other methods to detect differentially expressed biomarkers have been devised. For example, Jia and Tseng [23] suggested an adaptively weighted (AW) statistic, and Song and Tseng [24] identified the *r*th ordered *p* value, rOP. By using meta-analysis-based methods, the statistical power (sensitivity) can be improved. In this study, we proposed an integrative deep learning method that adds a consolidating layer to the existing deep learning method. We also used the backpropagation algorithm to update the weights in the integrative deep learning. We applied the lasso penalty to the objective function for parameter estimation. In order to evaluate the performance of the proposed method, we conducted a simulation study to compare it with the performances of the meta-SVM and metalogistic regression based on the Youden J index. We observe that integrative deep learning is robust for the variance of data. Furthermore, integrative deep learning even performs well when there is noise in the datasets that do not have any signal gene among the three datasets. Generally, the simulation results of integrative deep learning are stable. We also conducted real data (TCGA) analysis. Jia and Tseng [23] mentioned that they only considered combining multiple microarray studies and that it can be extended to combinations of multiple genomic, epigenomic, and/or proteomic studies. As we use the three different types of data, we accomplished the extended study. Based on the results of gene set enrichment analysis, we obtained that TCGA multiple omics data involve significantly enriched pathways which contain information related to breast cancer-like ABC transporter, CAMs. Overall, the results of real data analysis are consistent with existing biological truth. Therefore, they show that the proposed method, the integrated deep learning, can discriminate signal genes from the nonsignal genes.

## Figures and Tables

**Table 1:** Simulation results of metalogistic regression, meta-SVM, and the integrative deep learning (no inclusion of random study).

Methods	*σ*	Sensitivity (s.e)		Specificity (s.e)		Youden J index
Meta-SVM	0.1	1	(0)	0.9859	(0.001)	0.9859
	0.3	1	(0)	0.1597	(0.007)	0.1597
	0.5	0.9930	(0.0006)	0.1498	(0.006)	0.1491
	0.7	0.9777	(0.0003)	0.5399	(0.007)	0.5100
	0.9	0.9999	(0)	0.0135	(0.001)	0.0120

Metalogistic regression	0.1	0.068	(0.0017)	1	(0)	0.0680
	0.3	0.2000	(0.006)	1	(0)	0.2000
	0.5	0.2937	(0.0089)	0.9814	(0.0029)	0.2715
	0.7	0.3027	(0.01)	0.9484	(0.005)	0.2512
	0.9	0.3006	(0.012)	0.9085	(0.005)	0.2092

Integrative deep learning	0.1	0.7502	(0.02)	0.845312	(0.02)	0.5973
	0.3	0.6625	(0.026)	0.7817	(0.02)	0.4427
	0.5	0.7208	(0.024)	0.6911	(0.024)	0.4119
	0.7	0.7042	(0.026)	0.6625	(0.022)	0.3666
	0.9	0.7000	(0.03)	0.6427	(0.029)	0.3427

**Table 2:** Simulation results of metalogistic regression, meta-SVM, and the integrative deep learning (inclusion of 1 random study).

Methods	*σ*	Sensitivity (s.e)		Specificity (s.e)		Youden J index
Meta-SVM	0.1	0.8514	(0.008)	0.9531	(0.003)	0.8045
	0.3	0.7804	(0.009)	0.8502	(0.005)	0.6345
	0.5	0.8868	(0.007)	0.4567	(0.008)	0.3435
	0.7	0.9930	(0.002)	0.0208	(0.002)	0.0138
	0.9	0.8465	(0.012)	0.4899	(0.013)	0.3364

Metalogistic regression	0.1	0.1347	(0.007)	0.9392	(0.002)	0.0739
	0.3	0.2131	(0.007)	0.9548	(0)	0.1680
	0.5	0.2638	(0.01)	0.9338	(0.003)	0.1977
	0.7	0.2555	(0.01)	0.8965	(0.004)	0.1520
	0.9	0.2652	(0.01)	0.8706	(0.004)	0.1359

Integrative deep learning	0.1	0.7708	(0.024)	0.8345	(0.02)	0.6052
	0.3	0.6812	(0.026)	0.7302	(0.023)	0.4114
	0.5	0.6708	(0.022)	0.6989	(0.022)	0.3697
	0.7	0.6979	(0.027)	0.6395	(0.029)	0.3375
	0.9	0.7583	(0.028)	0.5828	(0.028)	0.3411

**Table 3:** Simulation results of metalogistic regression, meta-SVM, and the integrative deep learning (inclusion of 2 random studies).

Methods	*σ*	Sensitivity (s.e)		Specificity (s.e)		Youden J index
Meta-SVM	0.1	0.8284	(0.008)	0.9815	(0.001)	0.8099
	0.3	0.9090	(0.006)	0.1453	(0.005)	0.0543
	0.5	0.9990	(0.001)	0.0010	(0.0003)	0
	0.7	0.8518	(0.013)	0.2736	(0.012)	0.1254
	0.9	0.9944	(0.0017)	0.0056	(0.001)	0

Metalogistic regression	0.1	0.1423	(0.008)	0.9062	(0.003)	0.0485
	0.3	0.1861	(0.01)	0.9145	(0.003)	0.1006
	0.5	0.2319	(0.01)	0.8678	(0.004)	0.0977
	0.7	0.2527	(0.01)	0.8170	(0.006)	0.0697
	0.9	0.2583	(0.01)	0.8359	(0.005)	0.0942

Integrative deep learning	0.1	0.7666	(0.018)	0.9135	(0.012)	0.6802
	0.3	0.7562	(0.023)	0.7338	(0.02)	0.4901
	0.5	0.7208	(0.022)	0.6968	(0.018)	0.4177
	0.7	0.7187	(0.03)	0.6192	(0.029)	0.3380
	0.9	0.7770	(0.02)	0.5229	(0.03)	0.3000

**Table 4:** The brief descriptions of the three data information used in real genomic application.

ID	Study	Type	# of samples	Control (ER+)	Case (ER−)	Reference
TGCA-BRCA	Breast cancer	mRNA	300	234	66	The Cancer Genome Atlas (TCGA)
TGCA-BRCA	Breast cancer	Methylation	300	234	66	The Cancer Genome Atlas (TCGA)
TGCA-BRCA	Breast cancer	CNV	300	234	66	The Cancer Genome Atlas (TCGA)

**Table 5:** Selected features of multiple omics data (TCGA) via the integrative deep learning.

Three multiomics data of breast cancer (TCGA)
ABCA3 ABCA6 ABCC8 ABCG1 ACOT4 ACP5 ACSM1 ADAM8 ADRA2A AEBP1
AGR2 ALDH2 ASPN BCAS4 BNIPL C12orf54 C1orf64 CALB2 CAPSL CCDC80
CD36 CLDN3 CLDN8 CXCL13 CYP21A2 DACH1 DEFB1 DKK2 DNALI1 DSC2
DYX1C1 ENPEP ERP27 ESR1 ESRRG FAM3D FRK GBP1 IGFALS IL22RA2
LRRN2 OXGR1 PEX11 A PTH2R ROR2 SELE SORBS2 SPATA18 SYT9 TBC1D9
TBX21 TCN1 TFAP2B TNIP3 TOB1 TSPYL5 VCAN VGLL1 ZFP2

^*∗*^This gene set is significantly enriched in the ABC transporters and CAMs (KEGG) (ABC transporters: *p* value=8.489*e* − 07; Cell adhesion molecules (CAMs): *p* value=8*e* − 03).

## Data Availability

The Cancer Genome Atlas (TCGA) including mRNA, copy number variation (CNV), and epigenetic DNA methylation is available at http://cancergenome.nih.gov. In addition, our integrative deep learning R package (DeepOmics) is posted online at SungHwan Kim's website (https://sites.google.com/site/sunghwanshome/) and github (https://github.com/JaYeonLIm/Integrative-deep-learning-for-identifying-differentially-expressed-DE-biomarkers).

## Supplementary Materials

Supplementary Materials Detailed information about the R package and post hoc analysis (previously ascertained genes and coexpression networks) related to identified biomarkers is given. 1: R package deep omics. Table S1: these genes are not biomarkers but still potential biomarkers. Figure S1: gene networks using String tool.
