# Supplementary material for: Integrative Deep Learning for Identifying Differentially Expressed (DE) Biomarkers
Source: Comput Math Methods Med. 2019 Nov 2;2019:8418760. doi: 10.1155/2019/8418760 (PMC6935456; doi:10.1155/2019/8418760)
Supplement: Supplementary Materials — Detailed information about the R package and post hoc analysis (previously ascertained genes and coexpression networks) related to identified biomarkers is given. 1: R package deep omics. Table S1: these genes are not biomarkers but still potential biomarkers. Figure S1: gene networks using String tool. [file 8418760.f1.pdf]

Supplementary material: Integrative deep learning for identifying differentially expressed(DE) biomarkers

Jayeon Lim[1] Jiyeon Kim[4] Cheolyong Park[4] Junsang Cho[3] SoYoun Bang [2] and SungHwan Kim[1]

[1]*Deparment of Applied Statistics, Konkuk University, Seoul, South Korea*
[2]*Deparment of DataScience, Konkuk University, Seoul, South Korea*
[3]*Industry-University Cooperation Foundation, Konkuk University, Seoul, South Korea*
[4]*Department of Statistics, Keimyung University, Daegu, South Korea*

June 19, 2019

1 R package Deep omics

We implemented source codes for deep learning analysis. On our end, our integrative deep learning R package is posted up online at author's webpage.
(https://sites.google.com/site/sunghwanshome/)

2 Table S1

Our model selects 59 genes mostly associated with breast cancer and of which 13 genes have not reported as biomarkers for breast cancer. Even so, they can be still viewed as potential biomarker candidates, in the sense that they are predominantly focused on cancer-related biomarkers. Notably Table S1 shows 13 gene symbols. PEX11A in Table S1 is known to significantly associate with risk of lung metastasis in ER- breast cancer (Morales et al. (2014)). Similarly, Britzen-Laurent et al. (2013) also discovered that GBP1 in Table S1 relates to the potential new therapeutic target for triple-negative breast cancer and a tumor suppressor in colorectal cancer cells. In future study, it is interesting to see that the mechanistic studies reveal biological underlying signals with respect to newly identified genes. The proposed model facilitates to perform exploratory analysis for sure.

Table S1 : These genes are not biomarkers but still potential biomarkers.
BNIPL C12orf54 CAPSL CCDC80 CLDN8 CYP21A2
DEFB1 ERP27 FAM3D GBP1 OXGR1 PEX11A SYT9

Table S 1

3 Figure S1

We reconstructed co-expression networks using String tool and compared with Figure 5 (networks created by Netbox). In Figure 5, we can see that the ESR1 gene is connected to many cancer related genes. To our surprise, ESR1 mutations have recently emerged as a key mechanism of AIs (Aromatase inhibitors) resistance in ER+ metastatic Breast cancer. (See Section 4.2,

page 16, line 2). Similarly, we also identified that connectivity of ESR1 is found highly intensive to diverse genes and leave this for future study.

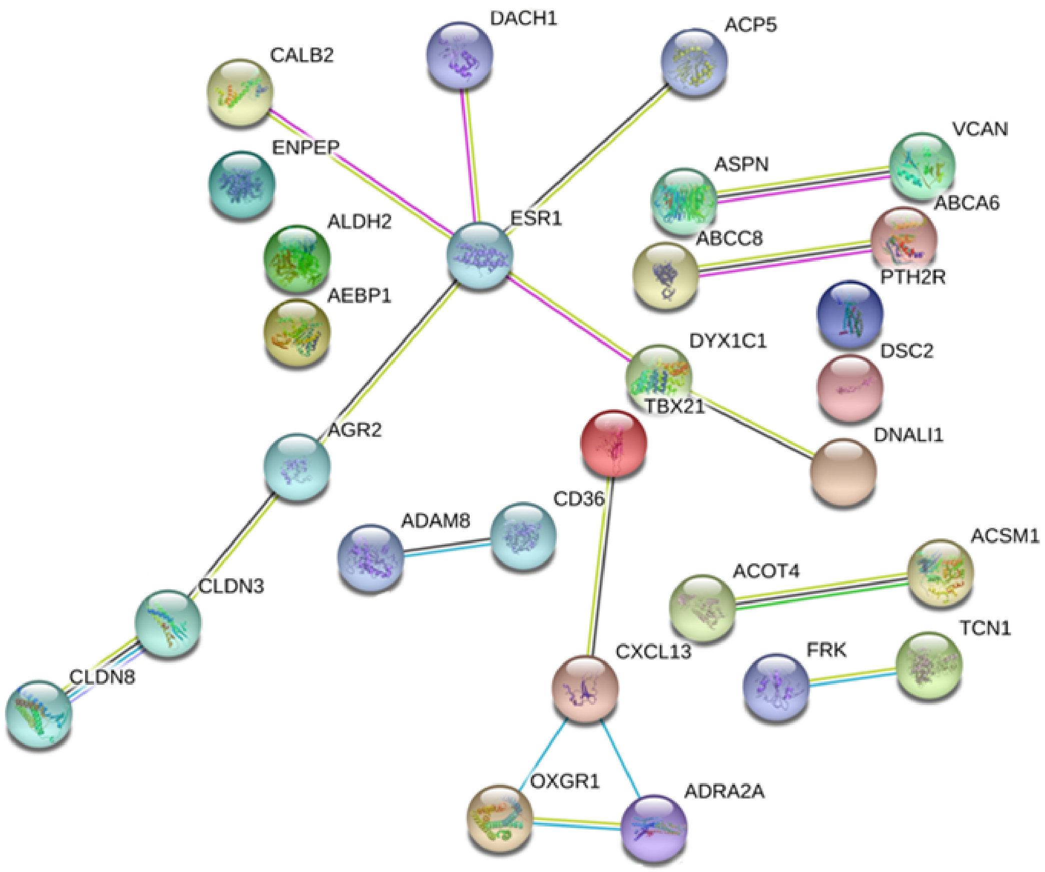

Figure S 1: Gene networks using String tool

References

Morales M, Arenas EJ, Urosevic J. (2015).suppresses breast cancer lung metastasis by regulating adhesion and differentiation. *EMBO Mol Med*, **6(7)**, 865-881.

Nathalie Britzen-Laurent, Karoline Lipnik, Matthias Ocker, Elisabeth Naschberger, Vera S. Schellerer, Roland S. Croner, Michael Vieth, Maximilian Waldner, Pablo Steinberg, Christine Hohenadl, Michael Strzl, et al. (2013). GBP-1 acts as a tumor suppressor in colorectal cancer cells. *Carcinogenesis*, **34(1)**, 153-162.
